# A Single Target Grasp Detection Network Based on Convolutional Neural Network

**DOI:** 10.1155/2021/5512728

**Published:** 2021-07-20

**Authors:** Longzhi Zhang, Dongmei Wu

**Affiliations:** State Key Laboratory of Robotics and Systems, Harbin Institute of Technology, Harbin 150001, China

## Abstract

Grasp detection based on convolutional neural network has gained some achievements. However, overfitting of multilayer convolutional neural network still exists and leads to poor detection precision. To acquire high detection accuracy, a single target grasp detection network that generalizes the fitting of angle and position, based on the convolution neural network, is put forward here. The proposed network regards the image as input and grasping parameters including angle and position as output, with the detection manner of end-to-end. Particularly, preprocessing dataset is to achieve the full coverage to input of model and transfer learning is to avoid overfitting of network. Importantly, a series of experimental results indicate that, for single object grasping, our network has good detection results and high accuracy, which proves that the proposed network has strong generalization in direction and category.

## 1. Introduction

Over recent years, deep learning has gained huge breakthroughs in computer vision [1, 2]. Unlike traditional hand-engineered features, deep learning can autonomic learning features from images, to acquire highly abstract and robust visual features via making use of image information to the most extent. Naturally, as one of the most representative deep learning models, convolutional neural network has become a research hotspot in computer vision, with easy training, high performance, few parameters, and strong generalization. Particularly, researchers have attempted to introduce it into research on robotic grasp detection, since its remarkable achievements in target detection [3–12].

Literature [13] innovatively used convolutional neural network for robotic grasps. More importantly, a deep neural network with four layers was proposed, which could effectively express multimodal features of grasping position, to achieve accurate detection of suitable grasping position on object [13]. Furthermore, a three-stage convolutional neural network was adopted to detect the grasping position of objects in depth image [14], where the first-level convolutional neural network was used for performing preliminary location of grasping position, the second-level convolutional neural network was utilized for acquiring the preselected grasping boundary, and the third-level convolutional neural network was to reevaluate the preselected grasping boundary. To perform operations, a two-step robotic grasp detection system was proposed [15].

Distinct from above thoughts, although convolutional neural network was also adopted to identify the grasping region of the object, the entire image of the object was taken as the input of network, to directly generate the position of the possible grasping region on object [16]. Reference [17] evaluated the possible position to be grasped of the target via predicting the grasping function learned from the convolutional neural network. In addition, researchers converted the grasp detection into an 18-channel binary classification [18] and adopted a convolutional neural network to learn the clamping rule of the two-finger gripper to obtain the optimal grasping position on the target. Xia et al. proposed a planar grasping pose detection method of the robot based on the cascaded convolutional neural network [19]; they established a cascaded two-stage convolution neural network model with position and attitude from coarse to fine to estimate the optimal grasping position and angle. In order to perform grasping new unknown model objects, visual feature points of an object in the process of being grasped were exacted via a convolutional neural network model, and a grasp strategy was constructed based on these visual feature points [20].

For actual robotic grasping, some grasp detection methods based on convolutional neural network models were put forward. Literature [21] proposed a hybrid deep architecture combining visual and tactile sensing for robotic grasp detection. An efficient framework of hierarchical cascaded forests to perform recognition and grasp detection of objects from RGB-D images of real scenes was proposed [22]. Ribeiro et al. [23] addressed the problems of grasp detection and visual servoing using deep learning and applied them as an approach to the problem of grasping dynamic objects. To acquire satisfactory grasp detection results, a self-supervised learning method was applied to learn grasping data directly collected by a robot [24]. To recognize and detect grasp rectangles on images of an object to be held by two-plates parallel grippers, a dictionary learning and sparse representation framework was proposed [25]. Also, unsupervised feature-learning methods were proposed for grasp detection [26–28]. In literature [26], a network model was proposed for predicting the 6 DOF pose of the target to confirm the position to be grasped. A beneficial attempt was conducted via using tactile sensors and an unsupervised feature-learning approach to predict whether a grasp is successful [27]. To clean water surface by aquatic robots, researchers came up with an unsupervised grasp detection method for water-surface object collection [28].

Additionally, for actual robotic grasping, another category of prediction approach is based on reinforcement learning. Zhang et al. proposed a reinforcement learning method for grasp detection to define a grasp as a point in a 2D image plane [29] via Q network [30] to perform target reaching after training in simulation. In literature [31], an asynchronous deep reinforcement learning approach was presented for learning robotic grasping policies, which can be trained on real physical robots. To perform complex sequences of pushing and grasping on a real robot, a method that combines deep reinforcement learning with affordance-based manipulation was put forward for detecting grasps [32]. Furthermore, to improve the flexibility of robotic detection for grasps, a curriculum-based reinforcement learning approach was conducted to learn reactive policies for the task of real picking [33]. Obviously, unlike above methods, grasp detection based on reinforcement learning mainly focuses on learning grabbing strategy for detecting grasps, rather than involving the network architecture itself.

However, with some success of grasp detection based on convolutional neural network in theories and applications, for grasp detection network inherence itself, overfitting in multilayer convolutional neural network still exists and leads to poor detection precision. To achieve highly accurate detection for grasps, a single target grasp detection network with high detection accuracy is proposed, which generalizes the fitting of angle and position.

The remainder of this paper is organized as follows.  Section 2 introduces our preliminary work to provide a theoretical basis for this research.  Section 3 gives an exhaustive formulation of our thoughts. Experimental results are shown in Section 4 to demonstrate the superiority of the proposed network. Ultimately, Section 5 concludes the paper and looks forward to the future work.

## 2. Related Work

### 2.1. Overview and Analysis of Components in Convolutional Neural Network

Objective of exploring each component in convolutional neural network is to deepen the understanding of network structure, so as to carry out our research. As a matter of fact, convolutional neural network is a feed-forward neural network, but distinct from ordinary neural networks, it is generally composed of a convolution layer, activation layer, pooling layer, and fully connected layer. Following, each component is overviewed and analyzed.

#### 2.1.1. Convolutional Layer

Convolutional layer is the core module in a convolutional neural network, which is usually composed of several convolution kernels with different sizes. After image input into the convolutional neural network, the convolution kernel performs convolution operations successively on the width and height of the image with a certain step length, to obtain a convolved feature vector.

Unlike connection ways of neurons in the ordinary neural network, convolution operation adopts sparse connection, which means that only neurons calculated with convolution kernels are connected to each other. Thus, this connection mode could increase the sparsity of the network to greatly reduce the number of network parameters and also could avoid overfitting of the network. In addition, convolutional neural network has the characteristic of weight sharing; that is, different positions of an image could be processed via the same convolution kernel, which could also reduce the number of network parameters.

Furthermore, the relation between input and output in a convolutional neural network is determined by convolution operation and selection of hyperparameters.

Assuming that the input image size is *H* × *W* × *C*, the convolution kernel size is *F* × *F* × *C*, the number is *N*, the convolution step is *S*, the unilateral filling size is *P*, and the output eigenvector is *H* × *W* × *C*, then the output could be expressed as

(1) $$\begin{array}{c} \left\{\begin{array}{c} H{}^{\prime }=\frac{H-F+2P}{S}+1,\\ W{}^{\prime }=\frac{W-F+2P}{S}+1,\\ C{}^{\prime }=N. \end{array}\right. \end{array}$$

Apparently, the output height and width of the convolutional neural network are determined by input, convolution kernel size, filling size, and step, while the output channel number is determined by convolution kernel number.

#### 2.1.2. Activation Function

Activation function plays an important role in the convolutional neural network. In fact, the inexistence of activation function will lead to the output that is a linear expression of input, which means that the network could only deal with linear problems, thereby greatly weakening the expression ability of the network model. As a result, to increase the nonlinear expression ability of network, activation function is usually added after convolutional layer.

Sigmoid function is one of the typical activation functions [34], and its expression is

(2) $$\begin{array}{c} \sigma \left(x\right)=\frac{1}{1+e^{-x}}. \end{array}$$

**Figure 1:**
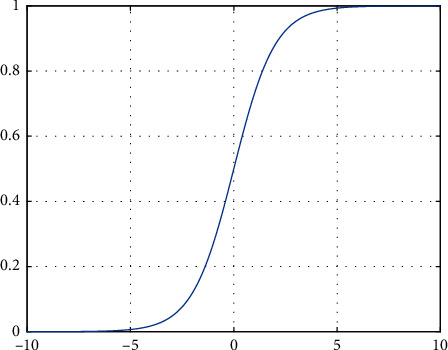
Sigmoid activation function.

In Sigmoid activation function, definition domain is (−*∞*, +*∞*), and value ranges at (0,1), as shown in Figure 1.

Sigmoid function was formerly widely used in the shallow neural network, but when the input is large, its gradient approaches 0, and with the increasing depth of the network, gradient dissipation is easy to occur in backpropagation, leading to failure of network training. Moreover, the output value of the Sigmoid function is not centered at 0.

Another typical activation function is the tanh function [35], which could be expressed as

(3) $$\begin{array}{c} \tanh\left(x\right)=\frac{e^{x}-e^{-x}}{e^{x}+e^{-x}}. \end{array}$$

**Figure 2:**
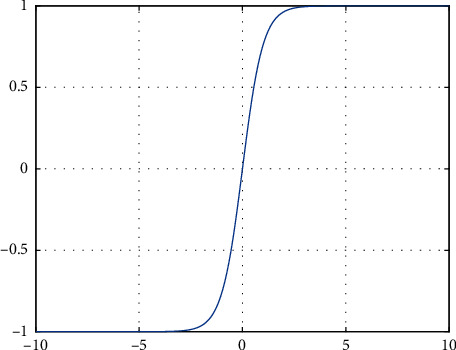
Tanh activation function.

Similar to the Sigmoid function, the definition domain of the tanh function is (−*∞*, +*∞*), and the value also ranges at (0,1). However, different from the Sigmoid function, the output value of the tanh function is centered at 0, as shown in Figure 2.

Although the output value of the tanh function is centered at 0, it still has not solved the problem that the network could not effectively backpropagate in case output or initial value is large. Hence, applications of above two activation functions tend to drop off.

Subsequently, a linear rectifier function called ReLU was proposed [36]; the expression is

(4) $$\begin{array}{c} f\left(x\right)=\left\{\begin{array}{cc} 0, & x<0,\\ x, & x\gg 0. \end{array}\right. \end{array}$$

**Figure 3:**
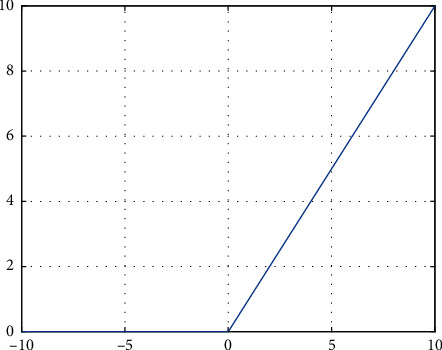
ReLU activation function.

ReLU function is simple and easy to derive, which does not increase the difficulty in process of backpropagation and greatly accelerates the training speed. Even though the function could not be differentiated at 0, it has left derivatives and right derivatives around 0 and any of them could be selected since values exactly falling at 0 are minor and hardly affect the overall results. The image of this activation function is shown in Figure 3.

In ReLU activation function, the gradient saturation phenomenon is inexistent and the gradient is always 1, leading to fast convergence. Simultaneously, there is low computation due to nonexponential operations. Furthermore, neurons with output less than 0 do not work, which greatly increases the sparse expression ability of the network, to improve the network generalization performance. Thus, ReLU activation function is most widely used in current deep neural networks.

#### 2.1.3. Pooling Layer

Pooling layer is also called downsampling layer and is commonly located behind the convolutional layer to reduce parameters number and computational complexity. Meanwhile, pooling layer could compress eigenvectors to exact main features and avoid overfitting. Generally, polling layer could compress the sizes of eigenvectors but could not change their depth.

**Figure 4:**
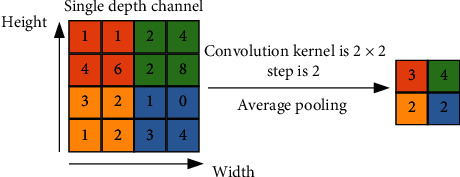
Calculation principle of average pooling.

**Figure 5:**
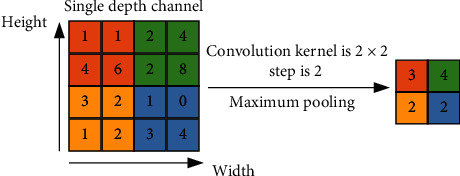
Calculation principle of maximum pooling.

Typical pooling methods include average pooling and maximum pooling; their calculation principles are, respectively, shown in Figures 4 and 5.

In Figures 4 and 5, the size of convolution kernels is the same, since above convolution kernel size is the most universally used in the convolutional neural network. It can be clearly seen that the average pooling takes the average value of convolution kernel region size and blurs the eigenvectors; thus, it is not conducive to feature extraction. However, maximum pooling takes the maximum value of convolution kernel region size and retains remarkable features. Accordingly, maximum pooling is mostly used at present.

#### 2.1.4. Fully Connected Layer

Fully connected layer is similar to the ordinary neural network, without weight sharing and sparse connection of convolutional layer, and each neuron in it is interconnected. In a convolutional neural network, the input of the fully connected layer is eigenvectors extracted from the convolutional layer, and the output layer is selected based on completed task, such as Softmax output layer and logistic regression layer.

However, fully connection gives rise to a large number of parameters. If the number of data is too small, the network will easily fall into overfitting. Thus, in convolutional neural network, the emergence of the fully connected layer is generally accompanied by the dropout layer. The dropout layer could stochastically discard some neurons to make them ineffective in fully connected layer. That is, the dropout layer is to imitate the sparse connection of the convolutional layer to prevent the overfitting of the network. In fact, the coefficient of dropout is confirmed by the specific application scenarios and network models, whose value is usually between 0.5 and 0.8 during training.

### 2.2. Performance Comparison of End-to-End Target Detection Algorithms Based on Convolutional Neural Network

Among target detection based on convolutional neural network, end-to-end networks directly detect the results from the image output, leading to a good performance in real time. Accordingly, we compare and analyze the performance of nowadays commonly used end-to-end networks to provide a theoretical foundation for our research.

In our implementation, VOC07 + 12 dataset is divided into a training set and test set, where the test set is 2007 test set, and the rest are training set. Detection results of different algorithms on test set are shown in Table 1.

It can be concluded from Table 1 that YOLOv2 is superior to YOLOv1 in accuracy and real time, and compared with YOLOv2-tiny, YOLOv2 gets a significant increase in accuracy at the expense of certain speed. Moreover, YOLOv2 is lower than SSD-300 in accuracy only at 1 percent, but more than four times faster in real time. Compared with SSD-512, YOLOv2 is 6.7 times faster than it, while being lower than it in accuracy only at 3.6%.

Through comparative analysis of above results, it can be seen that YOLOv2 has superiority over others in high accuracy and better real time. Hence, this paper introduces it into research on grasp detection and makes use of its end-to-end detection thought to conceive a single grasp detection network, which takes an image as input and grasp parameters as output. Also, the proposed network has a great generalization ability to fit in angle and position and has high detection accuracy.

## 3. Constructing Single Target Grasp Detection Network

### 3.1. Modeling Grasping Parameters

Indeed, the essence of grasp detection based on a convolutional neural network is to find grasping parameters that could achieve stable grasping. Hence, establishment of an appropriate grasping parameter model to achieve stable grasping is the key to the research of grasp detection based on a convolutional neural network.

Saxena et al. adopted a 2D grasping point as a grasp parameter model [37], and Le et al. utilized a pair of grasping points as a grasp parameter model [38]. However, the limitation of above grasp parameter models lies in that they could not fully represent the seven dimension parameters in grasping operation of the robot, and the other parameters need to be estimated separately.

Due to this, Jiang et al. proposed a seven-dimensional representation method combining 2D grasping rectangle and 3D point cloud [39], which described the 3D position, attitude, and size of the end-gripper. However, 3D point cloud data need to be calculated, which means that the extracted point cloud data require high precision and large amounts of computation.

To deal with above problem, Redmon and Angelova [16] simplified the model of literature [39]. Their contribution simplified the grasping in three-dimensional into planar grasping and proposed a five-dimensional parameter representation method based on the 2D grasping rectangle, which brought inspiration to our research.

Obviously, simplifying 3D grasping into 2D planar grasping and using a grasping rectangle to express the grasp parameters could effectively reduce the computation, and the issue of grasp detection becomes relatively simple. Particularly, the grasping rectangle is used to describe the grasp parameters, which makes grasp detection quite similar to object detection, while the distinction between the two is that the direction of the gripper needs to be considered in grasp detection.

Consequently, we utilize the strong learning ability of the convolutional neural network on image features to convert the grasp detection of the robot into target detection and adopt a 2D grasping rectangle to confirm the appropriate grasp parameters. More importantly, in order to enable 2D grasping to be fully mapped into 3D space and directly utilized by the robot to accomplish grasping operations, in this work, we assume that the gripper is always perpendicular to the *z*-axis to grasp vertically downward.

**Figure 6:**
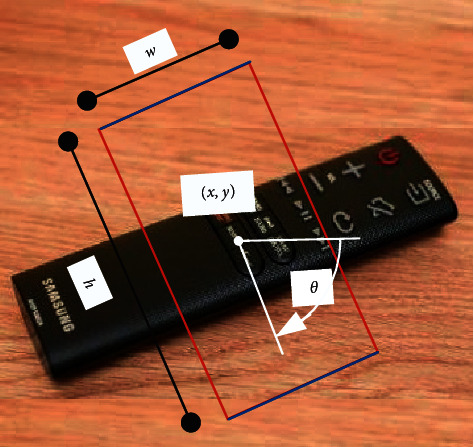
Schematic of grasp parameter model.

To sum up, we build up a model of grasp parameters with the manner of five-dimensional representation. More precisely, we use the position of the gripper (*x*, *y*), the direction of gripper *θ*, the opening size of the gripper before grasping objects *w*, and the size of gripper *h* to constitute a grasping rectangle, as exhibited in Figure 6.

The grasp parameters model could be expressed as

(5) $$\begin{array}{c} M=\left\{x,\;y,\;h,\;w,\theta \right\}, \end{array}$$

where (*x*, *y*) is the center coordinates of grasping rectangle, *θ* represents the rotation angle of grasping rectangle relative to the horizontal axis of the image (counterclockwise is positive), *w* means the width of grasping rectangle, and *h* refers to the height of grasping rectangle.

As displayed in Figure 6, a grasping rectangle of a remote device is composed of five grasp parameters defined by formula (5), where blue is on behalf of the gripper, red represents the distance between the two ends of the gripper before grasping, (*x*, *y*) is the center coordinates of grasping rectangle, and *θ* represents the rotation angle of grasping rectangle relative to the horizontal axis.

### 3.2. Modeling Grasp Detection Network

As mentioned above, YOLOv2 has obvious advantages in detection accuracy and real time. Thus, we introduce it into the research of grasp detection and utilize its “end-to-end” detection manner to establish a grasp detection network model with the proposed 5 grasp parameters as output. Accordingly, it is necessary to comprehend and analyze the network structure of YOLOv2 before modeling the grasp detection network.

**Figure 7:**
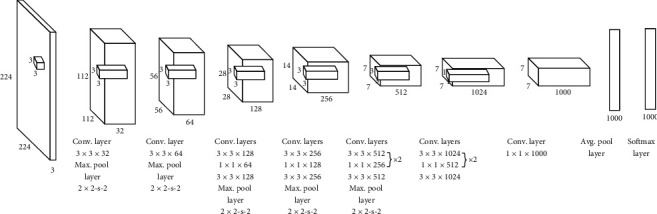
Network model of darknet19.

Darknet19 as the framework of YOLOv2 is composed of 19 convolutional layers and 5 maximum pooling layers. In darkent19, largely 3 × 3 convolutional kernels are used for feature exaction, and after each maximum pooling layer, channels are doubled to prevent information loss. Simultaneously, 1 × 1 convolutional kernels are added after 3 × 3 convolutional kernels to compress eigenvectors. Lastly, global average pooling is adopted to reduce dimension, and the Softmax layer is utilized for prediction. Furthermore, batch normalization is used for improving the stabilization and accelerating the convergence of the model in process of training. The network model of darknet19 is shown in Figure 7.

In fact, darknet19 has good performance in target detection, and the established grasp detection network model in this paper only needs the output 5 grasp parameters. Thus, in order to simplify the process of training network, meanwhile shortening the process of forward reasoning and backpropagation, thereby to avoid the occurrence of overfitting, we construct a grasp detection network model based on the network structure of darknet19, which has a relatively simple structure and could adapt to the proposed grasp parameters.

On the other hand, both accuracy and real time in grasp detection are taken into consideration; the constructed grasp detection network model should be able to make full use of powerful learning ability and extraction ability of convolutional neural network on image features and could avoid multiple time-consuming classification calculations in a small part of the whole image. Hence, the established grasp detection network model should be able to carry out bounding box regression on the whole image to acquire the appropriate grasping rectangle.

**Figure 8:**
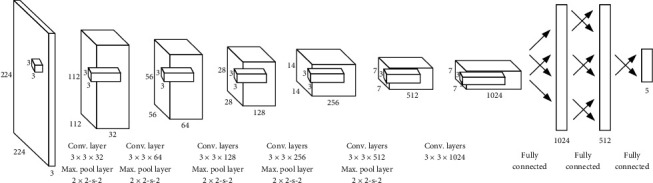
Structure of constructed grasp detection network model.

In summary, based on the network architecture of darknet19, we put forward a grasp detection network model with the whole image as input and five grasp parameters as output, whose structure is displayed in Figure 8.

As shown in Figure 8, compared with darknet19, the grasp detection network model established in this paper prunes the 1 × 1 convolutional kernel used for compressing eigenvectors, which was connected with 3 × 3 convolutional kernel, and removes the 3 × 3 convolutional kernel used for learning higher-level features, which was between 1 × 1 convolutional kernel and maximum pooling layer. The eigenvectors of 7 × 7 × 1024 are obtained after six convolutional layers and pooling layers and without connection of pooling layers behind the last convolutional layer. In addition, the 1 × 1 convolutional layer, fully connected layer, and Softmax output layer used for classification tasks are replaced by three fully connected layers with 1024, 512, and 5 neurons, respectively, where fully connected layers with 1024 and 512 neurons are used to deal with 7 × 7 × 1024 eigenvectors extracted by convolutional layer, and the last 5 neurons are used to output the grasp parameters.

When the original image is input into the network model, the convolutional layer is used to extract features from the image, and the fully connected layer of the last 5 neurons is used as the output layer corresponding to the coordinates of grasp parameters, where four neurons correspond to the position, width, and distance of the gripper. The grasping angle is symmetric; thus, *θ* ∈ (−*π*/2, *π*/2), but tan  *θ* is monotone increasing in this interval. Accordingly, the last neuron corresponds to the tan  *θ* value of the gripper relative to *z*-axis rotation angle. Although *θ* between (−*π*/2, *π*/2) is reasonable, tan  *θ* is closer to these two thresholds, and the value of |tan  *θ*| is greater, which is quite disadvantageous to the calculation of regression and even leads to difficulty in continuing training the network model. To avoid the emergence of this situation, we further limit the range of *θ*. Since tan ± 85° ≈ ±11, in this paper, the angle range is limited to *θ* ∈ (−85°, 85°), namely, only loss of 5°, and tan  *θ* is limited to a small range, which is convenient for regression calculation of the model.

Indeed, the constructed network model is for single object grasp detection; hence, each object only needs to predict once grasp. That is, as long as the image is input into the model, our model could directly make a global regression prediction of the image.

During training the proposed grasp detection network model, the model randomly selects a real value as a label to carry our regression with the predicted value. Since label value is always changing each time, the network model is uneasy to overfit in grasp parameters of an object. In order to better training the proposed network model, we define the loss function, which could be expressed as

(6) $$\begin{array}{c} F_{\text{coord}}=\lambda_{\text{coord}}\left({\left(x-\hat{x}\right)}^{2}+{\left(y-\hat{y}\right)}^{2}+{\left(h-\hat{h}\right)}^{2}+{\left(w-\hat{w}\right)}^{2}\right), \end{array}$$

(7) $$\begin{array}{c} F_{\text{angle}}=\lambda_{\text{angle}}{\left(\tan\;\theta -\tan\hat{\theta }\right)}^{2}\;, \end{array}$$

(8) $$\begin{array}{c} F_{\text{total}}=F_{\text{coord}}+F_{\text{angle}}, \end{array}$$

where *λ*_coord_ is the trade-off parameter of coordinate values losses, *λ*_angle_ is the trade-off parameter of angle values losses, *F*_coord_ is the coordinate values losses of the network, *F*_angle_ is the angle values losses of the network, and *F*_total_ is the total loss of network.

It can be seen from formula (6) and formula (7) that the paper adopts a sum of square errors to construct loss function, but different weight factors are used for different parameters to ensure that the contribution of each parameter to the loss is approximately consistent. Through statistics, rectangular center coordinates *x* and *y* are mostly between 100 and 150 pixels , as well as *h* and *w* are mostly between 20 and 30 pixels. Obviously, it is unreasonable to add directly and proportionately to the loss. Indeed, grasp position is quite important, but the opening and closing size of the gripper is also equally important. Hence, the regulator of coordinate values *λ*_coord_ is added before error losses of *x* and *y*, whose value is 0.1. Similarly, since the value of tan  *θ* is limited at the range of (−11,11), to adjust to the same level, the adjustment factor *λ*_angle_ is added before angular losses, whose value is 10. Through the above manners, losses of all parameters are basically guaranteed to account for the same proportion in total loss, which are conducive to the training network to obtain good results.

### 3.3. Selection and Preprocessing of Dataset

In order to verify the effectiveness of the proposed network model, it is necessary to select an appropriate dataset for the training model. At present, Cornell dataset is a widely used grasping dataset, which contains 240 common objects and 885 images obtained from different angles of these objects [1, 39]. In this dataset, numerous images contain the same kind of object, but the position and direction of the object in the image are different, which is extremely important for improving the robustness of the network model to the position and direction of the object during training. Consequently, this paper selects the Cornell grasping dataset to verify the validity of the proposed grasp detection network model.

However, in current data labels, cases that could not completely cover overall grasp positions and directions still exist. Thus, it is essential to preprocess the dataset to adapt the input of the model. In other words, we expand the dataset to achieve full coverage of input.

**Figure 9:**
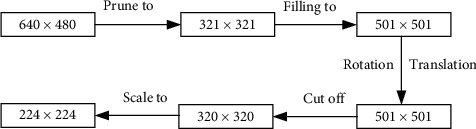
Flow of data preprocessing algorithm.

For the entire dataset, in order to prevent some objects in subsequent steps which are cut off, we primarily intercept pixel-sized areas of 321 × 321 from the center in each image and utilize a filling algorithm to fill in the neighboring pixels to pixel-sized areas of 501 × 501. Then we randomly spin the image five times with a certain angle. Namely, the image is randomly, respectively, moved five times within 100 pixels in *x* and *y* directions. Lastly, pixel-sized area of 320 × 320 from center in each image is cut out and scaled to the pixel-sized area of 240 × 240 that the network model needs to input. At the same time, label values also need to be synchronized to match the changes of each image. The whole process of the data preprocessing algorithm is shown in Figure 9.

After preprocessing, the dataset is expanded 125 times, including 110625 images, which satisfies the requirements of the following network training.

In our implementation, we use a 50-fold cross-validation method to test our model. Meanwhile, we adopt two ways to segment the image. The first one is to randomly segment all the images in the dataset, which means that the most likely occurrence of the test set is objects seen during training, but the direction is random and unseen. This image segmentation method tests the sensitivity of the network model to angle. The other is to randomly segment each category of the object in data; that is, all images of the same object are in the same cross-validation set, which means that objects in the test set are unseen during training, but the direction is seen. This segmentation manner has higher requirements and greater difficulty for the model, which is to test the generalization ability of the network model. In fact, generalization ability is exactly what we expect the proposed model should have.

### 3.4. PreTraining Grasp Detection Network

As a matter of fact, the dataset used in this paper contains a limited amount of data; directly training the network model easily leads to network overfitting. Yet pretraining a large-scale convolutional neural network model could greatly shorten training time and avoid overfitting [40]. Hence, it is essential to pretraining the network model to avoid overfitting during training.

Due to data similarity between grasp detection and target detection is high, and the training set has 88500 images after expansion of the whole dataset via preprocessing, whose amount is large. Thus, we could use transfer learning to extract image features from networks trained by datasets in target detection for grasp detection.

Nevertheless, transfer learning has different processing manners for diverse application scenarios. Consider that the only distinction between grasp detection and target detection is the output of grasp detection which has an extra gripper angle. Therefore, after data classification in the network, we use parameters of six convolution layers to send the extracted eigenvectors to the following fully connected layer for processing and predicting results. The three fully connected layers are trained from scratch and only one initialization value is given.

### 3.5. Training Grasp Detection Network

**Figure 10:**
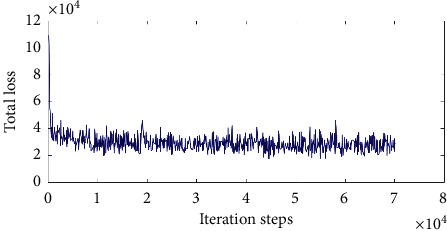
Changing curve of total loss.

After pretraining the network model, we adopt a small-batch gradient descent algorithm to training the network 100 times with the manner of end-to-end, where the value of each batch is 128. We set the learning rate *α* to 0.0005, the weight attenuation coefficient *λ* to 0.01, and the dropout parameter among three fully connected layers to 0.5. The loss of training processing is exhibited in Figure 10.

In Figure 10, the abscissa represents the number of training steps, and the ordinate refers to the corresponding loss value. Apparently, the total loss is decreasing with the increasing of iterative steps, but a short oscillation occurs when it decreases to a certain extent, and then it continues decreasing to a certain value, which indicates that the performance of the model for the training set tends to be stable at this time. Hence, in general, the model is reliable for the training set.

## 4. Experiments

### 4.1. Select and Determine the Evaluation Index of Proposed Grasp Detection Network

Point coordinates and rectangular coordinates are currently two general indexes to evaluate the performance of a grasp detection network [16, 41]. Indeed, point coordinates are to judge the quality of grasping via comparing the distance between the predicted coordinates of the center point in grasping rectangle and center points coordinates of all real grasping values, whereas this evaluation method does not consider the impact of grasping angle on accuracy, but angle value is particularly important in actual grasping. In addition, point coordinates also need to set another threshold to evaluate the results of point coordinates, which also affects the accuracy of calculation to a certain extent.

Rectangular coordinate is to judge the quality of grasping by comparing the difference between the predicted grasping angle and real grasping value. When the difference is less than 30° and the Jaccard similarity coefficient between the predicted grasping rectangle and real grasping value is greater than 25%, the grasping is considered to be effective [42]. In this paper, the Jaccard similarity coefficient is similar to Intersection-over-Union in target detection, which is defined as follows:

(9) $$\begin{array}{c} J\left(M_{g},M_{p}\right)=\frac{\left|M_{g}\cap M_{p}\right|}{\left|M_{g}\cup M_{p}\right|}, \end{array}$$

where *M*_*g*_ represents actual values of grasping rectangle and *M*_*p*_ refers to the predicted values of grasping rectangle.

Obviously, the value of the Jaccard similarity coefficient is larger, which indicates that the effect of grasp detection is better.

From above analysis, it can be concluded that the rectangle index considers both position and angle, which is more comprehensive than point coordinates and more convincing in judging the quality of grasping. Accordingly, in this paper, we adopt rectangle index to evaluate the performance of the proposed grasp detection network.

### 4.2. Experimental Results and Analysis

**Figure 11:**
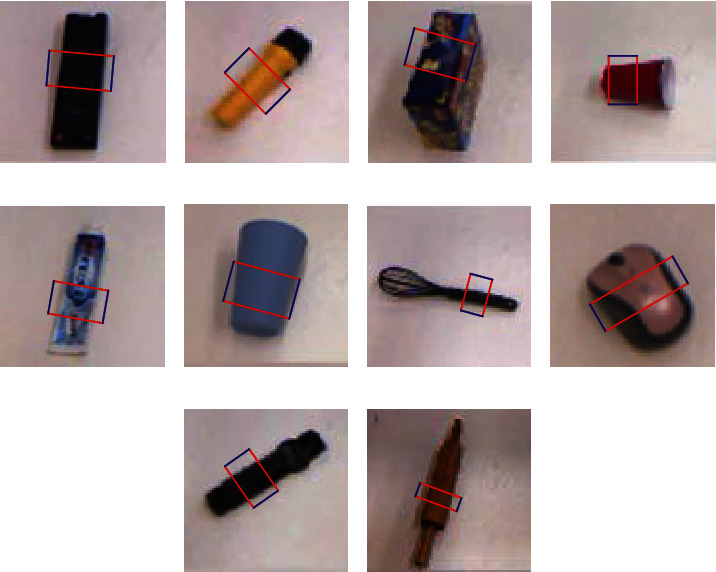
Some grasp detection results.

In order to validate the effectiveness of our network model, we conduct experimental verification on Cornell dataset. The image is input into the proposed network model, and the output result is the prediction grasping rectangle box of each input image. Some of the visual detection results are exhibited in Figure 11.

Obviously, detection results in Figure 11 illustrate that our model could detect the grasping region. Thus, to further illustrate the effectiveness of prediction, we calculate the Jaccard similarity coefficient of each prediction rectangle in Figure 11, and calculation results are shown in Table 2.

It can be clearly seen from Table 2 that all Jaccard similarity coefficients are greater than 0.25, which indicates that our grasp detection is effective, and grasp detection results for single object grasping could be regarded as good.

Through analysis of established network model, it can be known that acquired good detection results lie in two reasons. The first one is that our model adopts directly calculation of the loss and carry out global boundary regression on image to acquire the appropriate grasping rectangle. The other is that our model randomly selects a label value for each image during model training, which means that, after multiple training of dataset, the model predicts an average value for each object. Thus, for single object grasping, the predicted average value still has a good detection effect.

Additionally, to further verify the performance of the proposed network model, we make a comparison with other models based on convolutional neural networks, and the results are exhibited in Table 3.

It can be seen from above table that, in terms of detection accuracy, the prediction accuracy of our network model for image segmentation is 88.7%, and prediction accuracy for object segmentation is 87.2%; both of them stay at the third, belonging to an upper level. On the other hand, our research is inspired by literature [16, 39], and the comparison results in Table 3 show that our model is superior to the above two in detection accuracy, indicating that our research is meaningful even though it is not the best in above comparisons.

In summary, above experimental results demonstrate that the constructed network model has good detection results and high accuracy in single object grasping. Also, these results validate that our model is effective with strong generalization in direction and category.

## 5. Conclusions

In this work, a single target grasp detection network based on a convolutional neural network is put forward, which generalizes the fitting of angle and position with high detection accuracy. Specifically, we simplified 3D space grasping into 2D planar grasping and modeled grasping parameters with the manner of five-dimensional representation. Afterward, we adopted end-to-end detection ways to construct a grasp detection network model with the image as input and five grasping parameters as output. In order to verify the effectiveness of the proposed grasp detection network model, the Cornell grasp dataset is selected and expanded to match the input of the model. Furthermore, a 50-fold cross-validation method was adopted to test our network model, and the image was split into two ways. Moreover, for the sake of avoiding overfitting of the network in training, the constructed network model was pretrained via transfer learning. Ultimately, experimental results indicate that, for single object grasping, the proposed grasp detection network has good detection results and high prediction accuracy, which demonstrates that our detection model has strong generalization in direction and category.

Particularly, in the future, using other datasets to further optimize and validate our model is a beneficial work to be finished. Also, applying the proposed network to actual grasping operation is worth being deeply researched.

## Figures and Tables

**Table 1:** Detection results of different end-to-end algorithms.

Algorithm	Training set	Test set	mAP	FPS
YOLOv1	VOC07 + 12 trainval	VOC07 test	48.1	71
YOLOv1-tiny	VOC07 + 12 trainval	VOC07 test	33.5	282
YOLOv2	VOC07 + 12 trainval	VOC07 test	75.4	100
YOLOv2-tiny	VOC07 + 12 trainval	VOC07 test	41.1	250
SSD-300	VOC07 + 12 trainval	VOC07 test	75.5	18
SSD-512	VOC07 + 12 trainval	VOC07 test	79.0	15

**Table 2:** Jaccard similarity coefficients of grasp detection in Figure 11.

Image no.	Jaccard similar coefficient
a	0.83
b	0.85
c	0.82
d	0.51
e	0.81
f	0.68
g	0.85
h	0.75
i	0.86
j	0.67

**Table 3:** Grasping prediction accuracy of different algorithms on Cornell dataset.

Algorithms	Image segmentation accuracy (%)	Object segmentation accuracy (%)
Jiang et al. [39]	60.5	58.3
Lenz et al. [13]	73.9	75.6
Redmon and Angelova [16]	88.0	87.1
Wang et al. [15]	81.8	N/A
Guo et al. [21]	93.2	89.1
Asif et al. [22]	88.2	87.5
Ribeiro et al. [23]	94.8	86.9
Trottier et al. [25]	87.7	86.6
**Ours**	**88.7**	**87.2**

## Data Availability

In this paper, the dataset is the Cornell dataset.
